# Current hotspots and trends in cancer metabolic reprogramming: a scientometric analysis

**DOI:** 10.3389/fimmu.2024.1497461

**Published:** 2024-11-11

**Authors:** Shanshan Yang, Miaomiao Lin, Shaodong Hao, Hui Ye, Xuezhi Zhang

**Affiliations:** ^1^ Traditional Chinese Medicine and Integrative Medicine Department, Peking University First Hospital, Beijing, China; ^2^ Spleen and Stomach Disease Department, Fangshan Hospital, Beijing University of Chinese Medicine, Beijing, China

**Keywords:** metabolic reprogramming, cancer, bibliometrics, scientometrics, high-cited papers, hotspots

## Abstract

**Background:**

Metabolic reprogramming (MR) in cancer (CA) has been a focus of intense research in the recent two decades. This phenomenon has attracted great interest because it offers potential targets for cancer therapy. To capture the intellectual landscape of this field, we conducted a bibliometric analysis to assess the scientific output, major contributors, and trends in the MR/CA research.

**Methods:**

We performed a systematic search using the Web of Science to retrieve articles published on MR of cancer from 2006 until 2023. The bibliometric tools such as Biblioshiny, VOSviewer, and Microsoft Excel were used to identify the most prolific authors, institutions, citation patterns, and keywords. We also used co-citation analysis to map the conceptual structure of the field and identify influential publications. Furthermore, we examined the literature by analyzing publication years, citations, and research impact factors.

**Results:**

A total of 4,465 publications about MR/CA were retrieved. Publications on MR/CA increased rapidly from 2006 to 2023. *Frontiers in Oncology* published the most papers, while *Cell Metabolism* had the most citations. Highly cited papers were mainly published in *Cancer Cell*, *Nature*, *Cell*, *Science* and *Cell Metabolism*. China and the United States led the way in publications and contributed the most to MR/CA research. The University of Texas System, Chinese Academy of Sciences, and Fudan University were the most productive institutions. The profitable authors were Deberardinis Ralph J and Chiarugi Paola. The current topics included MR in tumorigenesis and progression of CA, MR of tumor cells and tumor microenvironment, the effect of MR on the CA treatment, the underlying mechanisms of MR (such as gene regulation, epigenetics, extracellular vesicles, and gut microbiota), and the modulation of MR. Some topics such as tumor microenvironment, lipid MR, circular RNA, long noncoding RNA, exosome, prognostic model, and immunotherapy may be the focus of MR/CA research in the next few years.

**Conclusion:**

This study evaluated the global scientific output in the field of MR/CA research, analyzing its quantitative characteristics. It identified some significant and distinguished papers and compiled information regarding the current status and evolving trends of MR/CA research.

## 1 Introduction

Cancer is a major global social, public health and economic problem, causing huge social and economic losses. There were close nearly 20 million new cancer cases and 9.7 million deaths from cancer in the year 2022, and according to the prediction, the number of new cancer cases worldwide may reach 35 million or more by 2050 (1). Therefore, it is very important to find the pathogenesis of cancer to prevent and treat cancer. Metabolic reprogramming (MR) of tumor cells is a hallmark of malignancy (2). Due to the scarcity of nutrients in the tumor microenvironment (TME), tumor cells must adopt a variety of metabolic adaptations and exhibit rapid adaptive responses to hypoxia and malnutrition conditions to meet their growth needs (3, 4), and this phenomenon of bioenergetic changes in tumor cells is known as MR of cancer (CA) (5). In the past two decades, due to the rapid development of cancer metabolism research, MR/CA research has received increasing interest and widespread attention (6). Multiple studies (2, 4, 7) have shown that MR plays a key regulatory role in the incidence, development, and treatment of cancer, and the modulation of MR may be an approach for preventing and treating CA.

Bibliometrics, the application of mathematics and statistical methods to books and other media of communication, offers a range of advantages for analyzing scholarly literature and research impact. Bibliometrics offers a means to assess the impact of individual research contributions, journals, and even entire institutions based on citation data and other indicators, enabling the processing of vast amounts of data that would be impractical to analyze manually (8). Over the last twenty years, exploring the intricate connection between MR and CA has gained significant momentum, resulting in an influx of groundbreaking studies that have garnered immense attention. However, there is currently no research on the quantitative investigation of MR/CA research. This paper conducts a visual representation and knowledge mapping of various bibliometric indicators, such as the prominent research areas, trending topics, and leading institutions that are active in the MR/CA domain to seek to provide scholars with a comprehensive understanding of the dynamic shifts and evolving trends in MR/CA research, ultimately facilitating a deeper grasp of the current research landscape and future directions.

## 2 Materials and methods

### 2.1 Data sources and search methods

The data sources for this study were the Science Citation Index Expanded (SCI-E) within the Web of Science Core Collection (WoSCC) database. In addition to providing abstracted and indexed records of articles, the Web of Science records citations made in scholarly publications, enabling researchers to track a publication’s citation history and determine which works are most influential within a given field. This facilitates searches for relevant literature based on keywords, authors, and other metadata.

**Figure 1:**
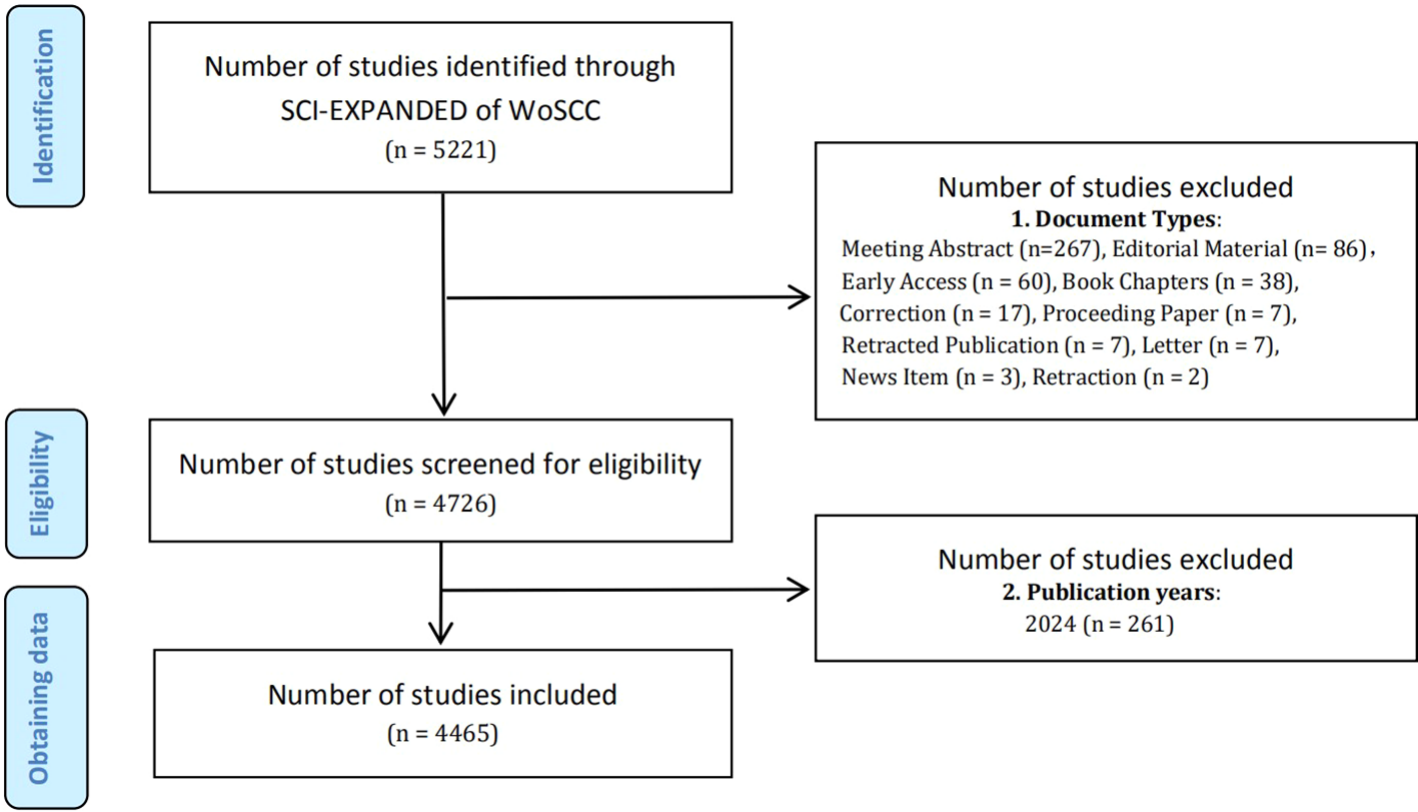
Flow chart of literature screening method in MR/CA.

On April 27, 2024 (Time Zone: East 8th District), all relevant search results were conducted and retrieved from the SCI-E within the WoSCC database. The terms “Metabolic Reprogramming” and “Cancer” as well as their synonyms from Medical Subject Headings (MeSH) in PubMed were used in the search, detailed in **Supplementary Material S1**. The criteria for selecting relevant studies encompassed: (1) publications dated between January 1, 2006, and December 31, 2023; (2) literary categories limited to “article” and “review”. As a result, a total of 4,465 papers were identified and recorded (**Figure 1**), comprising 2,843 articles and 1,622 reviews. The task of conducting the search and extracting data was independently undertaken by two researchers, SY and SH. We carefully refined the key information from the raw data and stored it in a text format for further analysis.

### 2.2 Data analysis and software applications

Bibliometric analysis often relies on specialized software tools to process and analyze the vast amounts of data involved. These tools provide functionalities for organizing, visualizing, and measuring the various aspects of scholarly production. Bibliometrix is an R package (version 4.1.3, the R Foundation) designed for performing bibliometric analysis. It offers a set of tools for the quantitative analysis of scientific literature, including citation analysis, co-citation analysis, and social network analysis. VOSviewer (version 1.6.20, Centre for Science and Technology Studies, Leiden University, The Netherlands) was used for creating maps based on bibliometric networks, such as citation, bibliographic coupling, and cooperation relationships. Microsoft Excel 2019 (Microsoft, Washington, USA) was used to extract and complete data processing and analysis. The content of bibliometric analysis encompasses several key areas: Publications analysis: Examining the number of publications (Np), publication types, and publication trends over time. Citation analysis: Assessing citation counts, citation impact, and the institutional distribution of citations. Author productivity: Analyzing the Np produced by the authors, countries, and institutions. Cooperation analysis: Investigating collaboration patterns among countries and identifying research networks. Journal impact analysis: Evaluating the influence and prestige of academic journals through metrics like the Journal Impact Factor (JIF). Citation Network analysis: Analyzes and visualizes citation networks to understand the citatory relationships between different documents. Trend analysis: Identifying emerging topics, research fronts, and paradigm shifts within a field.

## 3 Results

### 3.1 Annual scientific output

**Figure 2:**
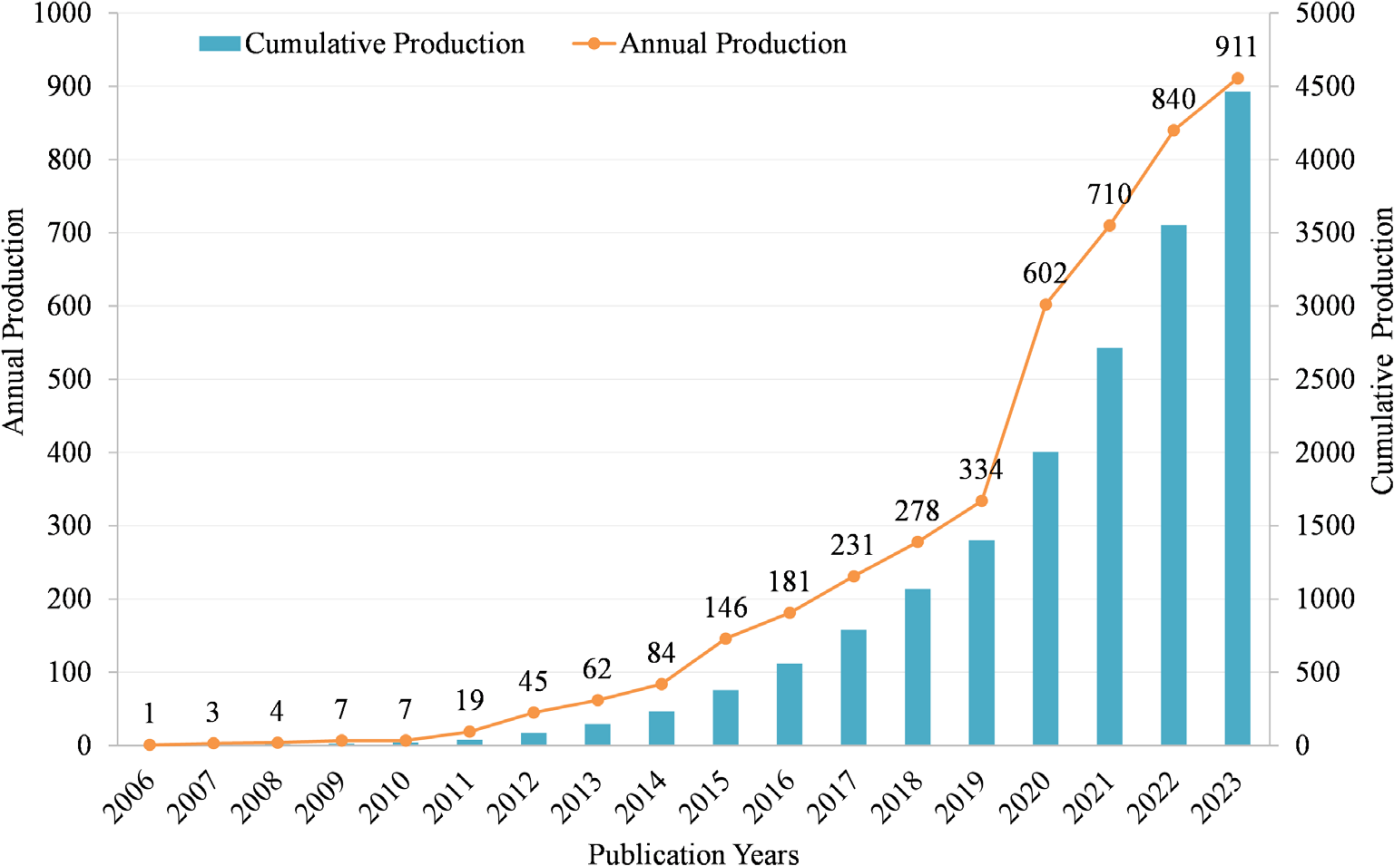
Annual and cumulative scientific output in MR/CA.

As shown in **Figure 2**, we can see that from 2006 to 2023, the annual Np is gradually increasing. In 2006, the Np was only 1, and by 2023, the Np increased to 911. Specifically, from 2006 to 2010, the Np remained relatively low, ranging from 1 to 7 per year. This indicates that the area was still in its early stages of exploration. Starting in 2011, the Np began to increase steadily, reaching 19 in 2011. This suggests that researchers were starting to recognize the potential significance of MR/CA. The growth rate accelerated significantly in the following years, with a sharp increase from 45 publications in 2012 to 84 in 2014. This rapid expansion indicates a growing interest and momentum in the field. From 2015 onwards, the Np continued to rise at a faster pace, reaching over 100 in 2015 and surpassing 200 in 2017. This rapid accumulation of knowledge indicates a maturing field with an increasingly large number of researchers exploring the intersection between MR and CA. In recent years, the growth rate has remained high, with over 600 publications in 2020 and over 900 in 2023. This suggests that the field is still expanding rapidly, attracting increasing researchers to contribute to the understanding of the MR in CA.

### 3.2 Main journals

**Figure 3:**
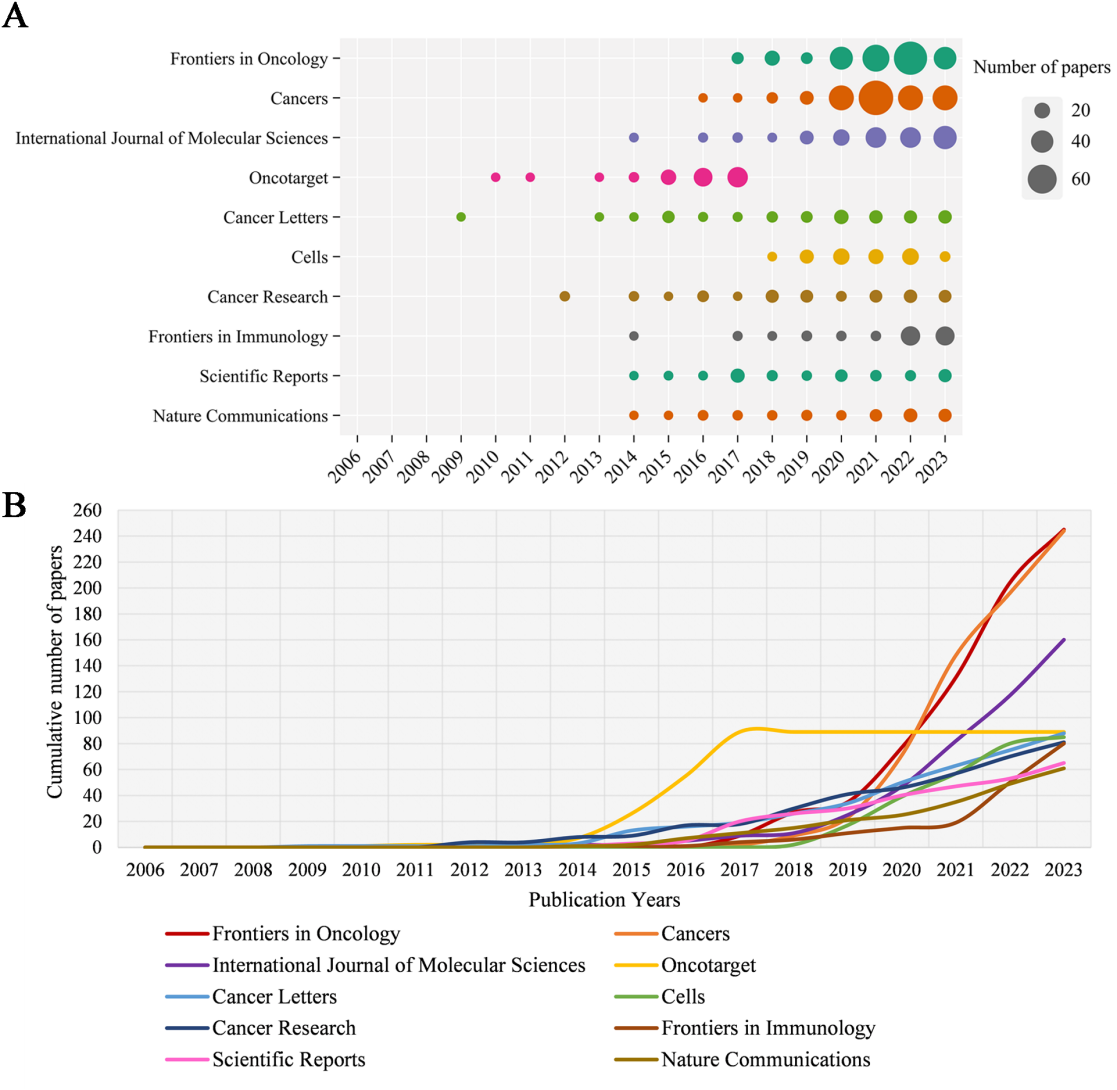
**(A)** Annual scientific output of the top 10 productive journals in MR/CA (the size of the circles represents the number of publications by each journal, with a larger circle indicating a higher annual output). **(B)** The cumulative scientific output of the top 10 productive journals in MR/CA. (the cumulative number of publications by the top 10 journals is represented by an upward-trending curve. The slope of the curve reflects the rate of accumulation, with steeper slopes indicating faster growth).

**Table 1** shows the top ten productive journals. *Frontiers in Oncology* ranks first with 245 articles and is the journal with the largest number of publications. *Cancers* followed with 244 published articles, taking second place. *International Journal of Molecular Sciences* published 160 articles, ranking third. **Figure 3A** shows the annual Np of the top ten journals. *Cancer letters* began to publish papers as early as 2009, and *Cancers* had the most publications in 2023. **Figure 3B** provides an overview of the combined output from these ten journals. With a total of 1,198 publications, these journals contribute approximately 26.83% of all research output, demonstrating their impressive productivity and influence in the field of MR/CA studies. The total citations (TC) serve as an indicator of the significance of a paper, while the H-index provides a means to assess its academic impact. **Table 2** shows the top ten most cited journals, with *Cell Metabolism* ranked first, followed by *Cancer Cell*, *Frontiers in Oncology*, *Cell* and *Cancer Research*. In the H-index, *Oncotarget* took first place, followed by *Cancer Research* and *Frontiers in Oncology*.

**Table 1:** The top 10 most productive journals in the field of MR/CA.

No.	Journals	Np	TC	H-index	2023IF	JCR	Countries
1	Frontiers in Oncology	245	5675	36	3.5	Q2	Switzerland
2	Cancers	244	4078	32	4.5	Q1	Switzerland
3	International Journal of Molecular Sciences	160	2979	30	4.9	Q1	Switzerland
4	Oncotarget	89	3933	40	–	–	USA
5	Cancer Letters	88	3261	32	9.1	Q1	Netherlands
6	Cells	85	2526	26	5.1	Q2	Switzerland
7	Cancer Research	81	4200	38	12.5	Q1	USA
8	Frontiers in Immunology	80	1255	16	5.7	Q1	Switzerland
9	Scientific Reports	65	1927	26	3.8	Q1	UK
10	Nature Communications	61	3425	32	14.7	Q1	UK

**Table 2:** The top 10 local impact journals in MR/CA.

No.	Journals	Np	TC	No.	Journals	Np	H-index
1	Cell Metabolism	27	9093	1	Oncotarget	89	40
2	Cancer Cell	16	7639	2	Cancer Research	81	38
3	Frontiers in Oncology	245	5675	3	Frontiers in Oncology	245	36
4	Cell	8	4617	4	Cancer Letters	88	32
5	Cancer Research	81	4200	5	Cancers	244	32
6	Cancers	244	4078	6	Nature Communications	61	32
7	Oncotarget	89	3933	7	Oncogene	61	31
8	Nature	16	3851	8	International Journal of Molecular Sciences	160	30
9	Nature Communications	61	3425	9	Cells	85	26
10	Nature Reviews Cancer	10	3360	10	Scientific Reports	65	26

### 3.3 Major countries/regions and institutions

**Figure 4:**
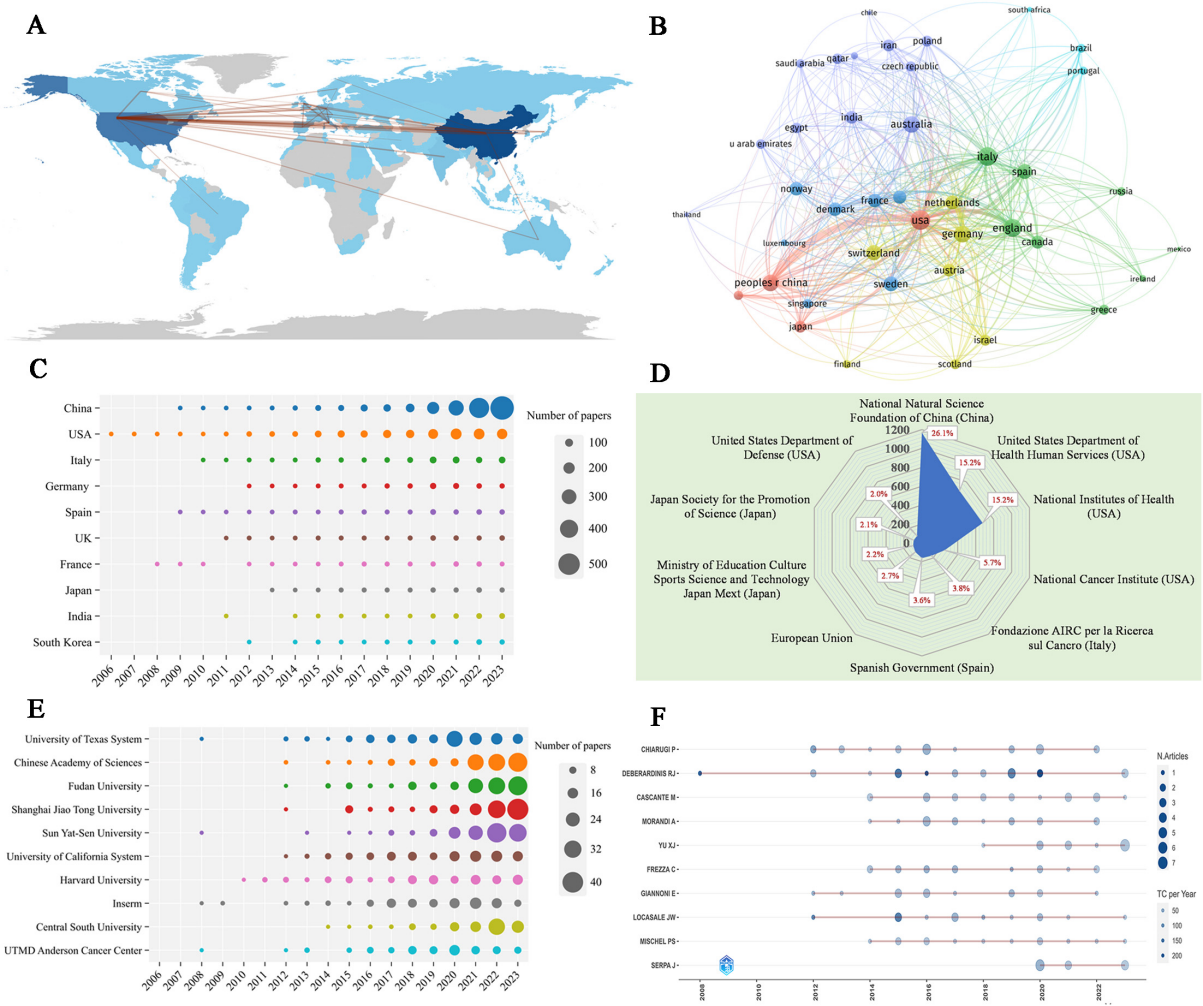
**(A)** Country scientific production and international collaboration network (min edges set to 10) in MR/CA. **(B)** Co-authorship network of the top productive countries in MR/CA (each node represents a country; each line represents a coordination relation and its thickness represents cooperation intensity). **(C)** Annual output of the top 10 productive countries over time in MR/CA. **(D)** The top 10 funding agencies and source countries in MR/CA research. **(E)** Annual output of the top 10 institutions over time in MR/CA. **(F)** Annual output of the top 10 productive authors over time in MR/CA (sizes of the circle signify scientific output, and the larger the circle, the more scientific output; the color depth of the circle indicates the annual citations, and the darker the color, the more citations).

**Table 3** shows the 10 most productive countries in MR/CA research. As we can see, China topped the list with 1,949 publications, followed by the United States with 1,234 publications (the TC and H-index were the highest), followed by Italy, Germany, Spain, the UK, and France from Europe. This shows that China and the United States occupied a leading position in MR/CA research, while some countries in Europe also show strong growth momentum. **Figures 4A, B** illustrate the scientific output of various countries and the primary national collaboration network within the field. Notably, the United States emerged as a leading force in international cooperation, maintaining a particularly close partnership with China. **Figure 4C** depicts the annual Np of the top ten countries. We can see that the United States issued the earliest and China developed the fastest. **Figure 4D** illustrates the main financial agencies. The National Natural Science Foundation of China (NSFC) toped the list with 1,164 projects. The U.S. Department of Health and Human Services (HHS) and the National Institutes of Health (NIH) ranked second and third, respectively, showing the support and investment of China and the U.S. in MR/CA research.

**Table 3:** The top 10 productive countries and institutions in MR/CA.

No.	Countries	Np	TC	H-index	No.	Institutions	Np	TC	H-index
1	China	1,949	48,603	99	1	University of Texas System (USA)	145	15,750	56
2	USA	1,234	80,828	134	2	Chinese Academy of Sciences (China)	141	5,611	39
3	Italy	391	15,160	65	3	Fudan University (China)	141	4,838	35
4	Germany	210	10,136	47	4	Shanghai Jiao Tong University (China)	139	3,361	32
5	Spain	191	8,068	46	5	Sun Yat-Sen University (China)	136	4,660	32
6	UK	177	10,107	54	6	University of California System (USA)	124	6,617	41
7	France	156	10,148	44	7	Harvard University (USA)	115	14,051	53
8	Japan	150	6,905	44	8	Inserm (France)	114	7,774	39
9	India	141	2,582	28	9	Central South University (China)	98	3,168	25
10	South Korea	130	4,778	38	10	UTMD Anderson Cancer Center (USA)	89	8,521	43

The top 10 most productive institutions in MR/CA are also displayed in **Table 3**. The institution with the largest Np was the University of Texas System, with 145 articles, followed by the Chinese Academy of Sciences and Fudan University, with 141 articles published respectively. Shanghai Jiao Tong University and Sun Yat-Sen University ranked fourth and fifth with 139 and 136 articles respectively. The University of Texas System and Harvard University ranked in the top two regarding TC and H-index. **Figure 4E** shows the annual Np of the top 10 institutions. As we can see, the MR/CA study was carried out earlier in 2008 by the University of Texas System, Inserm, UTMD Anderson Cancer Center, and Sun Yat-Sen University. Shanghai Jiao Tong University became the institution with the highest Np in 2023.

### 3.4 Main authors

**Table 4** shows the 10 most productive authors in MR/CA. They are mainly from the United States (n = 3), Italy (n = 3), China (n = 1), Germany (n = 1), Spain (n = 1) and Portugal (n = 1). The top five authors in terms of Np were Debsergatinis Ralph J, Chiarugi Paola, Cascante MARTA, Morandi, Andrea, and Yu Xianjun. Debsergatinis Ralph J, with 21 papers, a total citation of 8,668, and an H-index of 18, is affiliated with the University of Texas. Chiarugi Paola from the University of Florence had published 21 papers with 1,912 total citations and an H-index of 18. The third was Cascante MARTA with 19 papers, a total citation of 435, and an H-index of 11. Although Locasale Jason W published 14 articles, the number of citations ranked second, indicating the significant impact of his articles. **Figure 4F** shows the annual production of the top 10 authors. We found that Debsergatinis Ralph J began to study the earliest, while Yu Xianjun’s publication volume increased rapidly and he became the author with the highest Np in 2023.

**Table 4:** The top 10 productive authors in MR/CA.

No.	Authors	Np	TC	H-index	Affiliations	Countries
1	Deberardinis, Ralph J.	21	8,668	18	University of Texas	USA
2	Chiarugi, Paola	21	1,912	18	University of Florence	Italy
3	Cascante, MARTA	19	435	11	University of Barcelona	Spain
4	Morandi, Andrea	15	896	14	University of Florence	Italy
5	Yu, Xianjun	15	204	8	Fudan University	China
6	Locasale, Jason W	14	3,546	11	North Carolina State University	USA
7	Mischel, Paul S.	14	610	11	Stanford University	USA
8	Giannoni, Elisa	14	1,253	13	University of Florence	Italy
9	Frezza, Christian	14	1,392	11	University of Cologne	Germany
10	Serpa, Jacinta	13	231	8	Universidade Nova de Lisboa	Portugal

### 3.5 Analysis of cited papers in MR/CA research

#### 3.5.1 Top 20 most cited articles in MR/CA research

Highly cited papers are those that have been referenced frequently by other publications, which are pivotal contributions within the academic literature that have significantly influenced the landscape of a particular field. **Table 5** presents the top 20 highly cited original studies in MR/CA, mostly from *Cancer Cell* (n = 5), *Nature* (n = 3), *Cell* (n = 3), *Science* (n = 1), and their sub-journals. Browsing the relevant papers in MR/CA, we found that the reprogramming of tumor metabolism (mainly including glucose metabolism, fat metabolism, and amino acid metabolism) includes the MR of tumor cells and the MR of TME (including tumor-associated lymphocytes, fibroblasts, macrophages, natural killer cells).

**Table 5:** The top 20 most cited original research in MR/CA.

Rank	DOI Link	First author	Year	Journals	2023IF	JCR	TC
1	http://dx.doi.org/10.1016/j.cell.2012.01.058	Ying, HQ	2012	Cell	45.5	Q1	1385
2	http://dx.doi.org/10.1126/science.1218595	Jain, M	2012	Science	44.7	Q1	1033
3	http://dx.doi.org/10.1016/j.cell.2015.08.012	Ho, PC	2015	Cell	45.5	Q1	942
4	http://dx.doi.org/10.1016/j.ccr.2007.04.001	Zhang, HF	2007	Cancer Cell	48.8	Q1	693
5	http://dx.doi.org/10.1038/nature11743	Maddocks, ODK	2013	Nature	50.5	Q1	686
6	http://dx.doi.org/10.1016/j.ccr.2011.02.014	Finley, LWS	2011	Cancer Cell	48.8	Q1	624
7	http://dx.doi.org/10.1016/j.ccr.2013.01.022	Dong, CF	2013	Cancer Cell	48.8	Q1	589
8	http://dx.doi.org/10.1038/nature14587	Perera, R	2015	Nature	50.5	Q1	574
9	http://dx.doi.org/10.1038/s41556-019-0299-0	Chen, F	2019	Nat. Cell Biol.	17.3	Q1	454
10	http://dx.doi.org/10.1158/0008-5472.CAN-12-1949	Fiaschi, T	2012	Cancer Res.	12.5	Q1	412
11	http://dx.doi.org/10.1016/j.cmet.2014.06.004	Lee, JV	2014	Cell Metab.	27.7	Q1	397
12	http://dx.doi.org/10.1016/j.cmet.2015.08.007	Dupuy, F	2015	Cell Metab.	27.7	Q1	390
13	http://dx.doi.org/10.1038/ncomms4128	Nilsson, R	2014	Nat. Commun.	14.7	Q1	388
14	http://dx.doi.org/10.1038/nature12437	Dörr, JR	2013	Nature	50.5	Q1	381
15	http://dx.doi.org/10.1038/msb.2011.56	Gaglio, D	2011	Mol. Syst. Biol.	8.5	Q1	376
16	http://dx.doi.org/10.1016/j.ccell.2017.08.004	Zhang, Y	2017	Cancer Cell	48.8	Q1	368
17	http://dx.doi.org/10.1038/nn.3510	Flavahan, WA	2013	Nat. Neurosci.	21.2	Q1	355
18	http://dx.doi.org/10.1038/s41590-018-0251-7	Michelet, X	2018	Nat. Immunol.	27.7	Q1	352
19	http://dx.doi.org/10.1016/j.cell.2020.11.009	Ringel, AE	2020	Cell	45.5	Q1	318
20	http://dx.doi.org/10.1016/j.ccell.2017.06.004	Shukla, SK	2017	Cancer Cell	48.8	Q1	317

MR of tumor cells: The acquisition and utilization of nutrients are essential for tumor growth. For example, brain tumor-initiating cells (BTIC) can adapt to nutritional limitations by preferentially absorbing glucose. Specifically, BTIC shows a high level of Glucose Transporter 3 (GLUT3), which allows glucose to be preferentially absorbed, indicating that BTIC has a competitive advantage in relatively harsh microenvironments (9). Moreover, a 2012 study in *Science* conducted a large-scale cancer metabolic analysis and found cancer cells have a special preference for glycine, and the expression of glycine consumption and mitochondrial glycine biosynthesis pathway is closely related to the proliferation rate of cancer cells (10). A 2013 study in *Nature* showed that cancer cells rapidly use exogenous serine and serine deprivation triggers the activation of the serine synthesis pathway and rapidly inhibits aerobic glycolysis, leading to an increase in the flux of the tricarboxylic acid cycle (11).

MR of tumor microenvironment: A 2012 study (12) in *Cell* showed that there may be metabolic competition between T cells and tumor cells, CD4+ T cells within the tumor showed signs of glucose deficiency and diminished anti-tumor effector function. MR of T cells to increase phosphoenolpyruvate (PEP) production may enhance T cell-mediated antitumor immune responses. A 2020 study (13) found that tumors and CD8+ T cells showed different metabolic adaptations to obesity, tumor cells increase fat uptake under a high-fat diet (HFD), while tumor-infiltrating CD8+ T cells do not; These different adaptations lead to changes in fatty acid distribution in HFD tumors, thereby affecting CD8+ T cell infiltration and function; Blocking MR of tumor cells in obese mice may improve antitumor immunity. In addition, a paper found that enhancing the fatty acid metabolism of CD8+ T cells in a metabolically challenging tumor microenvironment can improve the efficacy of immunotherapy (14). A 2018 study published in *Nature* showed that the MR of natural killer (NK) cells in obesity limits anti-tumor responses and is the first to discover the molecular mechanism by which NK cells are blocked by excessive fat in obese individuals. It is pointed out that this ‘blockage’ does not prevent NK cells from recognizing tumor cells, but prevents them from killing tumor cells (15). A study (16) described the significance of glycolysis of cancer-associated fibroblasts (CAFs) on tumor growth and the phenomenon of lactic acid shuttle between CAFs and tumor cells. Lactic acid released by glycolytic tumor cells can up-regulate hypoxia-inducible factor-1 alpha (HIF-1α)-stabilizing long noncoding RNA (HISLA) in tumor-associated macrophages (TAMs), forming a feedforward loop between TAM and tumor cells. Blocking HISLA can inhibit glycolysis and chemotherapy resistance of breast cancer *in vivo* (17).

Some studies have explored the molecular mechanisms of MR. The KRAS gene is the most common carcinogenic gene. Oncogenic KRAS may maintain pancreatic cancer by regulating anabolic glucose metabolism (18). Glutamine may support the growth of pancreatic cancer through oncogenic KRAS-regulated metabolic pathways (19). The expression of oncogenic KRAS or AKT stimulates changes in histone acetylation, which precedes tumor development. The effect of AKT on histone acetylation is mediated by the metabolic enzyme ATP-citrate lyase, and the level of pAKT is significantly correlated with histone acetylation markers, indicating that acetyl-CoA metabolism is a key determinant of histone acetylation levels in cancer cells (20). In addition, the tumor suppressor p53 can promote cell survival during metabolic stress and transient activation of p53-p21 and cell cycle arrest promotes cell survival by effectively guiding the depleted serine stores to glutathione synthesis, thereby maintaining the cell’s antioxidant capacity (11). Transcription factor snails can cause MR, endowing tumor cells with cancer stem cell-like characteristics, and promoting drug resistance, tumor recurrence and metastasis. Snail-mediated inhibition of FBP1 loss provides a metabolic advantage for Basal-Like breast cancer (BBC), and the loss of FBP1 is a critical oncogenic event in epithelial-mesenchymal transition and BBC (21).

Mitochondrial deacetylase SIRT3 can mediate MR by destabilizing HIF-1α, a transcription factor that controls the expression of glycolytic genes. SIRT3 overexpression inhibits glycolysis and proliferation of breast cancer cells, while SIRT3 loss increases reactive oxygen species levels to induce tumorigenesis (22). HIF-1α target pyruvate dehydrogenase kinase 1 (PDK1) is required for liver metastasis, and HIF-1α activity and PDK1 expression are elevated in liver metastasis of breast cancer patients, indicating that PDK1 is a key regulator of breast cancer metabolism and metastasis potential (23). A study in *Nature* identified the MiT/TFE transcription factors as a major regulator of MR in pancreatic cancer and demonstrated that transcriptional activation of the clearance pathway gathered on lysosomes is a new marker of invasive malignant tumors (24). A study showed that the expression of metabolic enzymes highlights the key role of MTHFD2 and mitochondrial folate pathway in cancer, MTHFD2 is an integral part of mitochondrial one-carbon metabolism, a metabolic system recently associated with the rapid proliferation of cancer cells. RNA interference targeting MTHFD2 can lead to cancer cell death (25).

Targeted MR may become a promising method for anti-tumor therapy. *In vivo*, the drug targeting caused by therapy-induced senescence promotes tumor regression and further improves the treatment results, revealing the super catabolic properties of therapy-induced senescence, which can be treated by synthetic lethal metabolic targeting (26). A study in 2017 demonstrated that HIF-1α causes an increase in the glycolysis pathway and pyrimidine synthesis, which is the mechanism of gemcitabine resistance in pancreatic cancer, and targeting HIF-1α can increase the effectiveness of gemcitabine (27). Furthermore, glycolysis metabolite PEP may be a metabolic checkpoint for anti-tumor T-cell responses, and PEP carboxykinase 1 (PCK1) overexpressed T cells can limit tumor growth and prolong the survival time of melanoma mice. An acidic pH environment fosters tumor local invasive growth and metastasis, whereas oral administration of sodium bicarbonate effectively raises the peritumoral pH, thereby inhibiting tumor growth and invasion (28).

#### 3.5.2 Top 10 most cited reviews in MR/CA research

Highly cited reviews can provide synthesized overviews of a particular field of research and comprehensive analysis and insight into a topic by summarizing and discussing numerous primary studies. These review articles are highly valued for their ability to guide researchers through complex literature, clarify concepts, and propose new directions for future study. **Table 6** presents the list of the top ten most highly cited review articles published between 2008 and 2020.

**Table 6:** The top 10 most cited reviews in MR/CA.

No	Title	First author	Year	Journals	2023IF	JCR	TC
1	The Emerging Hallmarks of Cancer Metabolism	Pavlova, NN	2016	Cell Metab.	27.7	Q1	3484
2	The biology of cancer: Metabolic reprogramming fuels cell growth and proliferation	DeBerardinis, RJ	2008	Cell Metab.	27.7	Q1	2983
3	Metabolic Reprogramming: A Cancer Hallmark Even Warburg Did Not Anticipate	Ward, PS	2012	Cancer Cell	48.8	Q1	2307
4	Fundamentals of cancer metabolism	DeBerardinis, RJ	2016	Sci. Adv.	11.7	Q1	1795
5	Tumor cell metabolism: Cancer's Achilles' heel	Kroemer, G	2008	Cancer Cell	48.8	Q1	1713
6	Metabolic pathways promoting cancer cell survival and growth	Boroughs, LK	2015	Nat. Cell Biol.	17.3	Q1	1017
7	The PI3K-AKT network at the interface of oncogenic signaling and cancer metabolism	Hoxhaj, G	2020	Nat. Rev. Cancer	72.5	Q1	966
8	Metabolic reprogramming and cancer progression	Faubert, B	2020	Science	44.7	Q1	953
9	HIF-1 mediates metabolic responses to intratumoral hypoxia and oncogenic mutations	Semenza, GL	2013	J. Clin. Invest.	13.3	Q1	943
10	Lipid metabolic reprogramming in cancer cells	Beloribi-Djefaflia, S	2016	Oncogenesis	5.9	Q1	913

In 2008, a review (29) outlined that several core fluxes of MR/CA, including aerobic glycolysis, *de novo* lipid biosynthesis, glutamine-dependent supplementation, form a stereotyped platform that supports the proliferation of different cell types, and the regulation of these fluxes by cell mediators of signal transduction and gene expression, including PI3K/Akt/mTOR system, HIF-1 and Myc. The other review (30) discussed changes in signal transduction pathways and enzyme mechanisms that lead to MR of transformed cells and explained that in addition to the core role of HIF-1 activation, oncogenes (PI3K, Akt, Her2) and tumor suppressor genes (p53, VHL, PTEN, LKB1) also determine the MR of cancer cells at multiple levels. Likewise, the altered metabolism was caused by the active reprogramming of altered oncogenes and tumor suppressors, and the metabolic adaptation can be clonally selected during tumorigenesis (31). In addition, oncogenes and tumor suppressor genes play key roles in cellular metabolism, promoting MR and enabling cancer cells to acquire components from multiple metabolic pathways required for energy synthesis (32). HIF-1 mediates the metabolic response to intratumoral hypoxia and carcinogenic mutations, is activated in cancer cells through the loss of tumor suppressor function and the acquisition of oncogene function, and mediates metabolic changes that lead to cancer progression and therapeutic resistance (33).

In 2016, an article (6) provided an overview of six hallmarks of cancer-related MR, including disturbances in glucose and amino acid uptake, opportunistic patterns of nutrient access, biosynthesis and NADPH production using intermediates of the glycolytic/TCA cycle, increased nitrogen requirements, changes in metabolite-controlled gene regulation and interactions between metabolism and the TME. Meanwhile, a review (4) provided a conceptual framework to understand how and why MR occurs in tumor cells and what mechanisms link metabolic changes to tumorigenesis and metastasis. Another review (34) described the reprogramming of lipid metabolism in cancer cells and introduced the important role of specific lipids in mediating intracellular carcinogenic signal transduction, endoplasmic reticulum stress, and bidirectional crosstalk between cells of TME and cancer cells. In 2020, a paper (2) in *Science* showed that metabolic characteristics and preferences of tumors will change during cancer progression. Primary tumors and metastatic cancers have different metabolic characteristics even in the same patient or experimental model. A review (35) in *Nature Reviews Cancer* showed that the PI3K-AKT signal transduction network controls cancer cell metabolism by directly and indirectly regulating nutrient transport and metabolic enzymes, thereby linking oncogenic signaling and MR to support the survival and proliferation of cancer cells.

#### 3.5.3 Top 20 most cited references in MR/CA research

**Figure 5:**
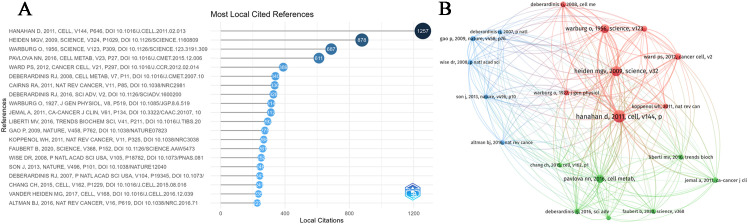
**(A)** The top 20 most cited references in MR/CA research. **(B)** Co-citation network of the top 20 most cited references in MR/CA research (The size of the circle represents the number of references).

**Figures 5A, B** illustrate the 20 most high-cited references and their respective citation interconnections. We found some important literature related to MR to understand its development process. As we can see, in 1927, German biochemist Warburg measured the changes of blood glucose in the inflow arteries and outflow veins of normal tissues and tumor tissues in animals and found that even in an environment with sufficient oxygen supply, tumor tissues were more inclined to obtain energy through glycolysis (36). In 1956, Warburg attributed this phenomenon to the mitochondrial dysfunction of cancer cells and proposed that the development of cancer cells is divided into two stages: the first stage is irreversible respiratory damage caused by many carcinogens and the second stage of cancer development is the result of the long-term struggle of injured cells to maintain their structure (37). In 2007, DeBerardinis et al. (38) found that in addition to aerobic glycolysis, transformed cells can participate in glutamine metabolism that exceeds protein and nucleotide synthesis requirements. In 2008, Wise et al. (39) found that Myc regulates the transcriptional program that stimulates mitochondrial glutamine decomposition and leads to glutamine addiction. In 2009, taking advantage of the high glucose uptake of tumors, Vander (40) developed a method (FDG-PET) for tumor diagnosis and therapeutic effect assessment and found that nutritional supplementation and strict glucose control were helpful in the treatment of tumors. Gao et al. (41) found that c-Myc inhibits miR-23a/b to enhance mitochondrial glutamine enzyme expression and glutamine metabolism. In 2011, Hanahan D and Weinberg RA (42) officially defined reprogramming of energy metabolism as one of the ten most important cancer criteria and a newly recognized hallmark of cancer. Cairns RA (43) summarized that the Warburg effect is regulated by PI3K, HIF, p53, MYC, and AMP-activated protein kinase (AMPK)-liver kinase B1 (LKB1) pathways. Koppenol et al. (44) reviewed Otto Warburg’s contributions to current concepts of cancer metabolism in detail. In 2013, Son et al. (19) showed that glutamine supported the growth of pancreatic cancer through the metabolic pathway regulated by KRAS. In 2015, Chang et al. (45) found that metabolic competition in the tumor microenvironment is a driver of cancer progression. In 2016, Liberti MV et al. (46) discussed in detail the historical perspective of the Warburg effect and several mechanisms of how the Warburg effect benefits cancer cells. Altman et al. (47) summarized the role of glutamine in cell growth and cancer cell biology. In 2017, Vander Heiden et al. (48) summarized the importance of cancer metabolism to cancer pathophysiology and clinical oncology. Changes in cell metabolism can promote transformation and tumor progression, and metabolic phenotypes can also be used to image tumors, provide prognostic information, and treat cancer.

### 3.6 Analysis of keywords in MR/CA research

Keyword analysis has important application value in bibliometric research. It can help researchers understand the content, structure, and characteristics of the literature more deeply, reveal the research topics and hotspots of the literature, evaluate the quality and value of the literature, and predict future research directions and hotspots.

#### 3.6.1 Most frequent words

**Figure 6:**
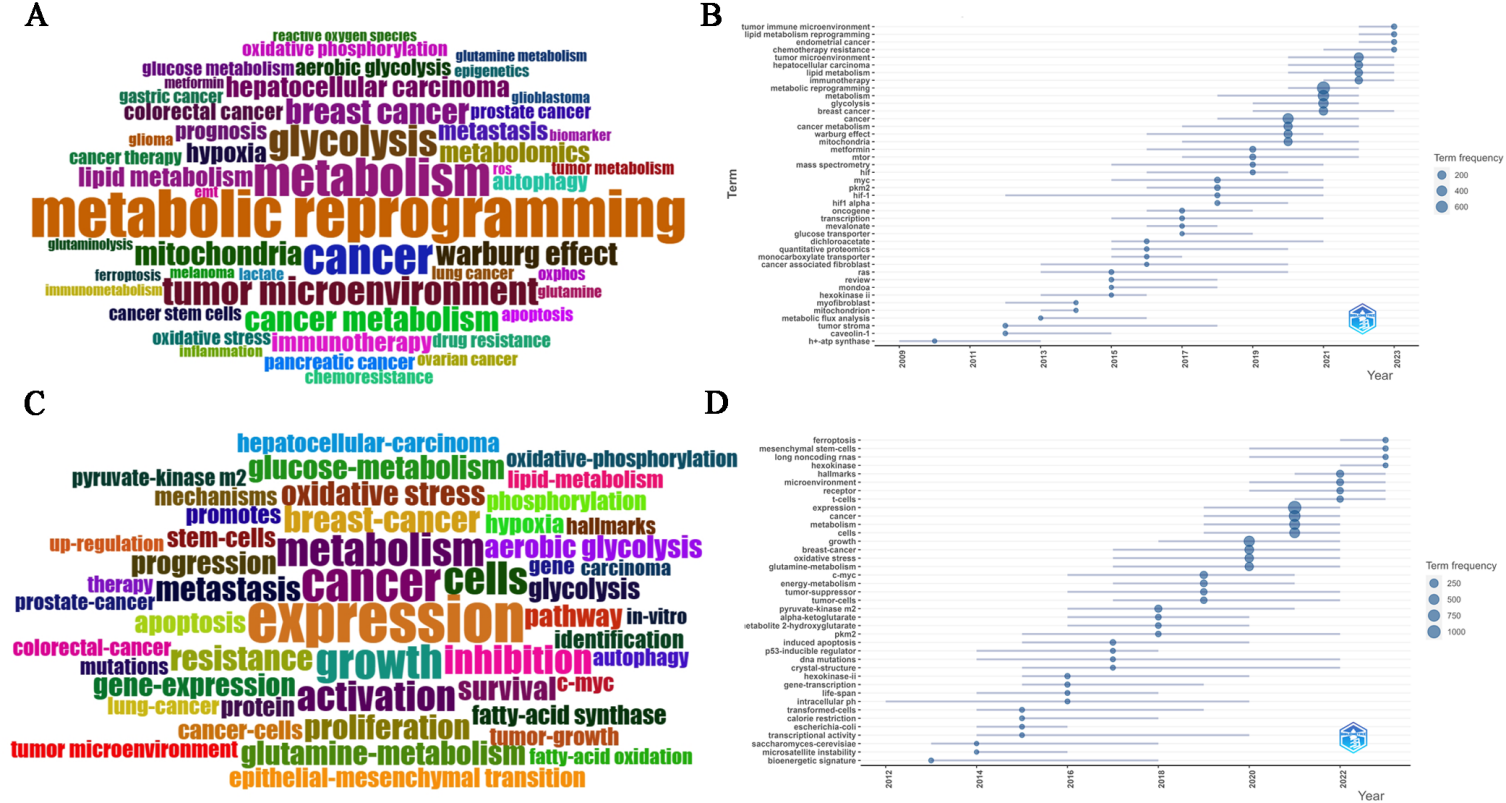
**(A)** Common author keywords in MR/CA research. **(B)** Evolution trends of common author keywords over time in MR/CA (The size of the circle represents the number of occurrences). **(C)** Common keywords plus in MR/CA. **(D)** Evolution trends of common keywords plus over time in MR/CA (The size of the circle represents the number of occurrences).

A total of 13,978 keywords were used in this study, comprising 6,545 author keywords and 7,433 additional keywords that were found in publications. The following author keywords (**Figures 6A**) were highly frequent: “metabolic reprogramming”, “metabolism”, “cancer”, “tumor microenvironment”, “breast cancer”, “hepatocellular carcinoma”, “colorectal cancer”, “glycolysis”, “aerobic glycolysis”, “cancer metabolism”, “mitochondria”, “Warburg effect”, “metabolomics”, “lipid metabolism”, “glucose metabolism”, “glutamine metabolism”, “metformin”, “hypoxia”, “epigenetics”, “immunotherapy”, “chemoresistance”, “drug resistance”, “cancer therapy”, “biomarker”, “prognosis” and “metastasis”. However, “expression”, “cancer”, “growth”, “metabolism”, “cells”, “activation”, “inhibition”, “breast-cancer”, “resistance”, “metastasis”, “pyruvate-kinase m2”, “mechanisms”, “oxidative stress”, “glutamine-metabolism”, “glucose-metabolism”, and “survival” were prominently keywords plus (**Figures 6C**).

#### 3.6.2 Trend analysis of keywords

At the same time, according to the keyword analysis, future research directions and hotspots can be predicted to support scientific research decision-making. As shown in **Figures 6B, D**, the main author keywords included “tumor immune microenvironment”, “lipid metabolism reprogramming”, “circular RNA”, “exosome”, “prognostic model”, “immunotherapy”, “lipid metabolism”, “glycolysis”, “tumor microenvironment”, “Warburg effect”, “metformin”, “mitochondria”, “myc”, “pkm2”, “hif-1” and “hif”. The main keywords plus included “c-myc”, “pkm2”, “long noncoding RNAs”, “ferroptosis”, “receptor”, “microenvironment”, “hallmarks”, “metabolism”, “cancer”, “oxidative stress”, “glutamine-metabolism”, and “tumor-suppressor”.

#### 3.6.3 Cluster analysis of keywords

**Figure 7:**
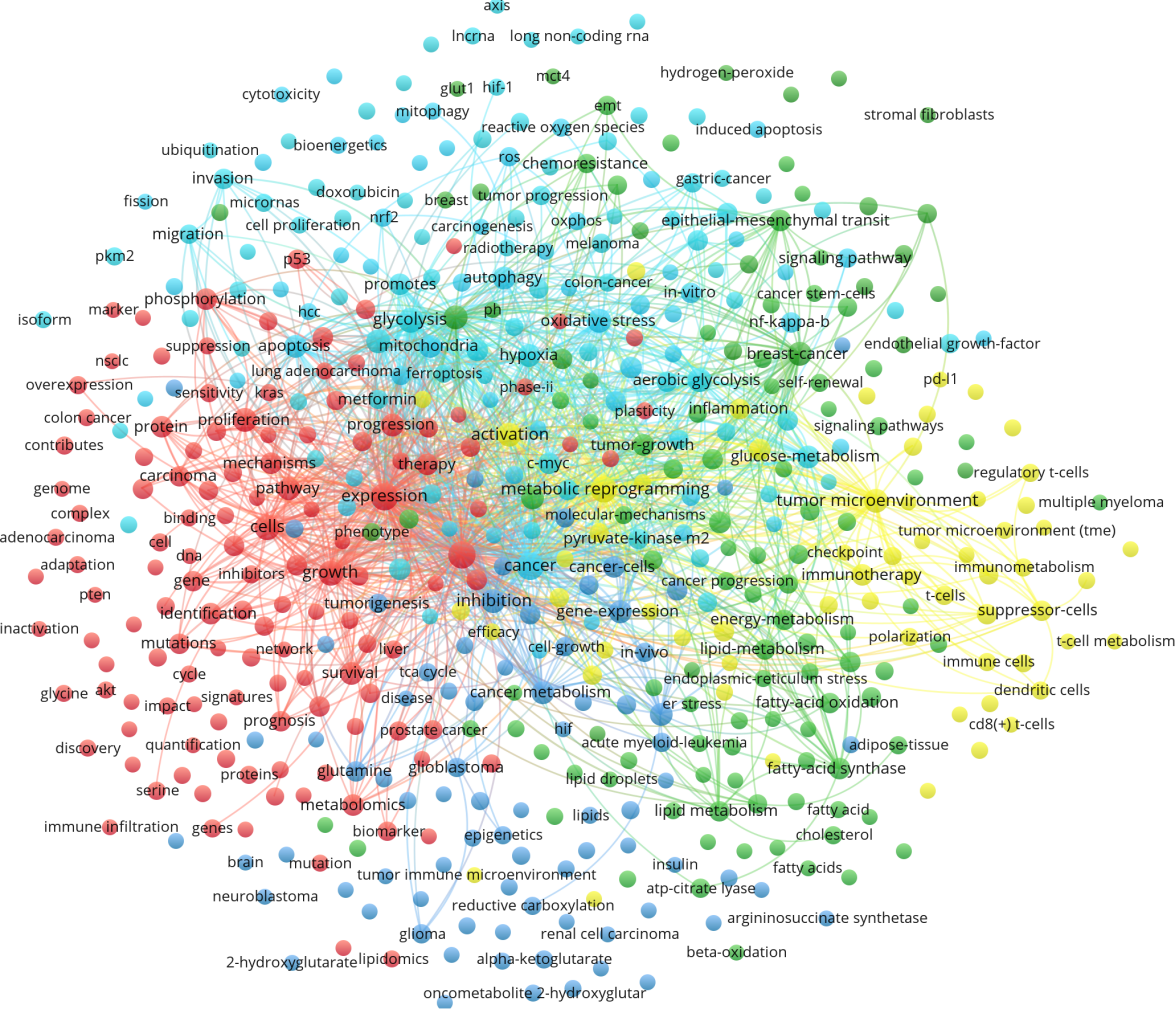
Cluster analysis of common keywords in MR/CA research (Nodes are represented by different colors, and edges show the relationship between them by connecting them, the size of the nodes represents the frequency of occurrence, different colors represent the results of different cluster analysis in the network diagram).

**Figure 7** shows different colors representing the results of different cluster analyses in the network diagram. These clustering results are usually grouped based on the similarity of the relationship between small dots. The following is a description of the clustering results of different colors:

(1) Red clustering: this mainly represents processes closely related to the mechanisms of energy metabolism, including “genes”, “proteins”, “expression”, “overexpression”, “metabolomics”, “mechanisms”, “p53”, “Kras”, “pten”, “myc”, “akt”, “Ras”, “EGFR”, “oncogenic Kras”, “proliferation” and “mutations”. These genes and proteins are related to tumor growth, invasion and immune escape and are important for cellular energy metabolism.
(2) Dark blue clustering: this is related to “cancer metabolism” such as “glutamine metabolism”, “amino acid metabolism”, “serine metabolism”, and “one-carbon metabolism”. It also includes “isocitrate dehydrogenase 1”, “tumorigenesis”, “idh2 mutations”, “alpha-ketoglutarate”, “2-hydroxyglutarate”, “oncometabolite 2-hydroxyglutar”, “TCA cycle”, “reductive carboxylation”, and “transporters”, which are associated with the biological process of mitochondrial metabolism. Glutamate is primarily formed from alpha-ketoglutarate, an intermediate in the TCA cycle.
(3) Green clustering: this focuses on “lipid metabolism reprogramming”, including keywords such as “energy-metabolism”, “fatty-acid synthase”, “fatty-acid oxidation”, “lipid metabolism”, “lipid droplets” and “cholesterol-metabolism”. Meanwhile, it also pays attention to “cancer stem cell”, “EMT”, “endoplasmic-reticulum stress”, “glut1”, “therapy resistance”, “chemoresistance”, “chemotherapy resistance”, “cisplatin resistance”, “gemcitabine resistance”, “multidrug resistance” and “drug resistance”. This may indicate that metabolic reprogramming affects anti-tumor therapy.
(4) Light blue clustering: this focuses on specific metabolic pathways in MR and tumor progression. This cluster may contain “glucose metabolism”, “glycolysis”, “Warburg effect”, “long non-coding RNA”, “lncRNA”, “circular RNA”, “microRNA”, “microRNAs”, “pyruvate-kinase m2”, “pkm2”, “HIF-1”, “sirtuins”, “c-Myc”, “signaling pathway” and “hypoxia”. In addition, some keywords focus on mitochondrial-related research, such as “mitochondria”, “oxidative stress”, “autophagy”, “reactive oxygen species”, “ROS”, “apoptosis”, “mitochondrial dysfunction”, “mitophagy”, “nrf2”, “nf-kappa-b”, “DNA-damage” and “oxidative phosphorylation”.
(5) Yellow clustering: this mainly focuses on the TME and tumor immune microenvironment, and keywords such as “immune metabolism” and “tumor microenvironment” appear as nodes and are linked to words such as “immunosuppression”, “immune evasion”, “immunotherapy”, “PD-L1”, “tumor-associated macrophages”, “macrophage polarization”, “regulating t-cells”, “t-cells”, “suppressor-cells”, “t-cell metabolism”, “immune cells”, “CD8(+) t-cells”, and “gut microbiota”.

## 4 Discussion

In the process of abnormal proliferation, to obtain more energy to support autologous proliferation, invasion, and metastasis, cancer cells usually need to change their metabolic mode or increase their metabolic amount. Over the last two decades, with the escalating understanding of MR, there has been a growing body of research indicating that MR can influence the initiation and progression of cancer as well as modify the outcomes of tumor therapy. Consequently, studies exploring the interrelationship between MR and CA have gained momentum, leading to numerous significant research advancements. As a result, this study conducted a bibliometric analysis to furnish researchers with a fundamental understanding of the status and evolving trends in the cross-disciplinary intersection between MR and CA.

### 4.1 Analysis of document issuance in MR/CA

Overall, the Np shows a stable growth trend. This suggests that there may be an increase in research interest, activity, or publishing capacity in this field. This growth may be due to the expansion of the research field, the increase of scientific research funds, the increase in the number of researchers, the improvement of publishing channels, or other related factors. We try to divide this duration into the following periods: The initial period (2006-2010): The Np is relatively small but shows an increasing trend. Steady growth phase (2011-2018): The Np continued to grow steadily. Accelerated growth phase (2019-2020): Growth is more pronounced, possibly due to some breakthroughs in research areas. Stable high growth period (2021-2023): The Np reached a higher level and appeared to be stable.

The Np can be used as an indicator to measure the activity of journals in this field, but it does not directly reflect the quality or influence of journals. In general, indicators such as impact factor (IF) and H index (H-index) are more commonly used to evaluate the academic influence of journals. Our study showed that the *Frontiers in Oncology*, *Cancers and International Journal of Molecular Sciences* ranked among the top three in the Np, the *Oncotarget* had the highest H-index, and *Cell Metabolism* had the highest TC. The top 20 highly cited articles were mainly published in *Nature*, *Cell*, *Science*, and *Cancer Cell*. The impact factors of these articles are all above 10, and all of them are JCR zone 1.

Primarily originating from China and the United States, these publications are also contributed by Italy, Germany, and Spain. However, it is noteworthy that China still has room for improvement in MR/CA-related research and could benefit from strengthening international collaborations. Among the top ten institutions, those from China, the United States, and France demonstrate their strong research productivity in this field. University of Texas System (the highest TC), University of California System, and Harvard University (the secondary high TC) from the United States produced many papers. In China, the Chinese Academy of Sciences, Fudan University, and Shanghai Jiao Tong University published the most articles and made important contributions to MR/CA research.

The top ten authors were mostly from world-class research universities. The author with most Np, the highest TC and the H-index was Deberardinis RJ from the *University of Texas*, who had made great contributions to the study of MR/CA and published a large number of highly cited papers (4, 29, 32) and high-IF papers in *Nature* (49, 50), *Science* (2), *Nature Medicine* (51), and *Nature Reviews Cancer* (52), especially paid attention to the effect of MR on tumorigenesis and progression of CA, oncogenes and tumor suppressors and metabolic pathways in MR. In the last few years, he has increasingly focused on targeting tumor metabolism to improve anti-tumor efficacy (53). Chiarugi P from the *University of Florence* has long been interested in the role of cancer-associated fibroblasts (CAFs) in MR (16, 54, 55) and microRNA regulator of MR (56, 57), and his articles are mainly published in *Cancer Research* and the *Oncotarget*. Locasale JW from *North Carolina State University* had the second-highest TC and published many highly cited and high-IF papers in *Cell* (12, 18), focused on the role of gene regulation (18), epigenetics (7, 58, 59), and extracellular vesicles (60) in MR/CA.

### 4.2 Hotspots and Frontiers in MR/CA research

Through a cluster analysis of commonly used keywords and highly cited articles, hotspots and boundaries in MR/CA research have been delineated. This study found that the current hot topics of MR/CA focus on five perspectives: (1) the effect of MR on tumorigenesis and progression of CA; (2) MR in tumor cells and tumor microenvironment; (3) The effect of MR on the treatment of CA; (4) the underlying mechanisms of MR; (4) Modulating MR for the prevention and treatment of CA. In addition, new research priorities such as tumor microenvironment, reprogramming of lipid metabolism, circular RNA, long non-coding RNA, exosome, prognosis model, and immunotherapy are not only the current hotspots but also are anticipated to remain the focus of MR/CA research for the next few years.

#### 4.2.1 The effect of MR on tumorigenesis and progression

Some studies have shown that the MR of glucose, fatty acids, and amino acids may provide nutrients to support cancer cell growth and promote cancer progression.

##### 4.2.1.1 Glucose metabolic reprogramming

The Warburg effect (aerobic glycolysis) affects cancer cells through increased glucose uptake and glucose fermentation to lactic acid (46). By restricting mitochondrial oxidative metabolism, the Warburg effect aids cancer cells in minimizing oxidative stress, which in turn facilitates metastasis and dissemination (61). For example, pancreatic cancer (PC) cells show extensive enhancement of glycolysis, including overexpression of glycolytic enzymes and increased lactic acid production, using glucose MR to meet their energy needs and support malignant behaviors (62). The glycolysis process of PC cells produces many substrates and promotes the growth and metastasis of tumor cells through the interaction of glycolytic core enzymes and actin, thereby supporting tumor growth (18).

##### 4.2.1.2 Fatty metabolism reprogramming

Tumor cells actively or passively remodel lipid metabolism, using the function of lipids in various important cellular life activities to evade treatment attacks (63). Lipid metabolism regulates a variety of oncogenic signaling pathways involved in tumor initiation, development, invasion, and metastasis (64). For example, the protein tyrosine phosphatase receptor type O inhibits the tumorigenesis and progression of colorectal cancer by modulating the metabolism of fatty acids (65). Adipocyte and lipid MR plays a role in supporting cancer growth, metastasis, and drug resistance (66). Fatty acid synthase (FASN)-mediated *de novo* fatty acid synthesis contributes to the functional maturation of Treg cells, and the absence of FASN in Treg cells inhibits tumor growth (67).

##### 4.2.1.3 Amino acid metabolism reprogramming

Amino acid derivatives contribute to epigenetic regulation and immune response related to tumor occurrence and metastasis (68). For instance, glutamine is a conditional essential amino acid with a wide function. Increased glutamine catabolism is a key feature of cancer cell metabolic characteristics, promoting the core metabolism of proliferating cells by supporting energy production and biosynthesis (69). Glutamine may support the growth of pancreatic cancer through the KRAS-regulated metabolic pathway (19). In addition, Jain et al. (10) found that glycine plays a key role in promoting the growth of tumor cell lines. Liu et al. (70) found that the MR of proline and glutamine contributes to the proliferation and metabolic response regulated by the oncogenic c-Myc.

##### 4.2.1.4 Mitochondrial metabolic reprogramming

Warburg proposed that mitochondrial respiratory impairment is a prerequisite for the malignant transformation of cells, and aerobic glycolysis and mitochondrial dysfunction have been widely accepted as hallmarks of tumors. However, recent studies have revealed that mitochondrial metabolism is essential for tumor growth, and mitochondrial MR is a dynamic process in tumor development, and its metabolic flexibility can meet the different needs of tumors at various stages from tumorigenesis to metastasis. In mitochondrial metabolism, the isocitric dehydrogenase 1 (IDH1) is involved in the citric acid cycle, converting isocitrate to α-ketoglutarate (α-KG), while mutant IDH 1 converts α-KG to 2-hydroxyglutarate (2-HG) to be involved in tumorigenesis.

##### 4.2.1.5 Key enzymes/regulatory factors in MR

Metabolic enzymes are the direct executors of the metabolic regulation of tumor cells, driving the MR of tumor cells. Concretely speaking, pyruvate kinase M2 (PKM2) is a restrictive glycolytic enzyme and a key regulatory molecule in the aerobic glycolysis of tumors. Enhanced PKM2 activity can induce glycolytic MR of cancer stem cells and promote gastric cancer progression (71). PDK1, a key regulator of MR, is elevated in liver metastasis of breast cancer patients and can promote tumor cell proliferation and migration by enhancing the Warburg effect (23). Glucose transporter 1 (GLUT1) inactivation leads to MR for oxidative phosphorylation, generating excess reactive oxygen species (ROS), and accumulated ROS enhances TNF-a-mediated tumor cell death (72). FASN promotes lymph node metastasis in cervical cancer via cholesterol reprogramming and lymphangiogenesis (73). Fatty acid-binding protein 5 (FABP5) may promote lymph node metastasis of cervical cancer by MR of fatty acid (74). STAT5A-dependent FABP5 expression plays a carcinogenic role in gastric cancer cells by reprogramming intracellular fatty acid metabolism (75). In addition, a study has shown that the amino acid transporter SLC7A5 is essential for the effective growth of KRAS mutant colorectal cancer (76). Glutaminase (GLS) driven glutamine catabolism may promote radiosensitivity of prostate cancer by regulating redox status, dryness, and ATG5-mediated autophagy (77).

#### 4.2.2 Tumor metabolic reprogramming: drivers and passengers

##### 4.2.2.1 MR of tumor cell: traditional perspective

MR of cancer cells refers to cancer cells adjusting and optimizing their internal metabolic processes to meet the demands of rapid proliferation and survival. This reprogramming enables cancer cells to utilize nutrients more efficiently, generate energy, and synthesize necessary biomolecules to support their growth and spread. MR of glucose, fatty acid, and amino acid in tumor cells provides an important material and energy basis for tumor growth, proliferation, invasion, and metastasis.

The “Warburg effect”, which promotes tumor cells to convert glucose into lactic acid under aerobic conditions, is one of the representative events in the autonomous regulation of MR in many malignant tumors and opens scholars’ research on MR in malignant tumors. Cancer cells have a special preference for glycine and the expression of glycine consumption and mitochondrial glycine biosynthesis pathway is closely related to the proliferation rate of cancer cells (10). Cancer cells rapidly use exogenous serine and serine deprivation triggers the activation of the serine synthesis pathway and rapidly inhibits aerobic glycolysis (11). In addition, cancer stem cells tend to form the smallest residual lesions after chemotherapy and exhibit metastatic potential through additional MR, leading to adaptive/acquired resistance to anti-tumor therapy (5). Glioblastoma stem cells can reprogram lysine catabolism to promote a state of tumor immunosuppression (78). Notably, some studies (2, 23) showed that primary tumors and metastatic cancers may have different metabolic signatures.

##### 4.2.2.2 MR of tumor microenvironment: emerging new paradigms

###### 4.2.2.2.1 MR of cancer-associated fibroblasts

CAFs can provide biomechanical support for tumor cells and promote epithelial-mesenchymal transition in primary tumors by secreting extracellular vesicles, increasing adhesion to tumor cells, remodeling the extracellular matrix (ECM) and altering their mechanical stiffness, which in turn promoting tumor metastasis (79). CAFs are the main regulatory factor shaping tumor metabolism, particularly through the dysregulation of multiple metabolic pathways. The configuration of these metabolic switches is postulated to influence diverse CAF behaviors and subsequently modify the behavior of tumor cells (80). Cancer-derived exosomal HSPC111 promotes liver metastasis of colorectal cancer by lipid MR in CAFs (81). CAFs play a role in the creation of ECM structure and the immune MR of the TME, thus affecting the adaptive resistance to chemotherapy (82).

###### 4.2.2.2.2 MR of tumor-associated macrophages (TAMs)

Immune cells show obvious metabolic changes that affect their immune function when they are activated. Both tumor cells and TAMs undergo MR to meet the energy demands of the TME. Understanding MR in TAM could elucidate immune escape mechanisms and provide insights into the repolarizing anti-tumor function of TAM (83). TAMs have different metabolic characteristics and are regulated by coordination factors and signaling pathways. After activation, classically activated M1 macrophages and alternatively activated M2 macrophages showed different patterns in glucose, lipid, amino acid, and iron metabolism (84). Typically, M1 macrophages rely primarily on aerobic glycolysis, whereas M2 macrophages rely on oxidative metabolism (85).

###### 4.2.2.2.3 MR of tumor-associated T lymphocytes

The MR of T cells is different from that of tumor cells (13). CD4+ T cells within the tumor showed signs of glucose deficiency and MR of CD4+ T cells to increase PEP production may enhance T cell-mediated antitumor immune responses (12). Enhancing the fatty acid catabolism of CD8+ T cells in a metabolically challenging TME can improve the efficacy of melanoma immunotherapy (14). The TME inhibits mitochondrial biogenesis of T cells and drives insufficient metabolism and dysfunction of T cells in tumors (86). The imbalance of amino acid consumption in immune cells may lead to impaired anti-tumor immunity. Amino acid metabolism in immune cells may enhance promising opportunities for cancer immunotherapy (87). Targeting lipid MR of immune cells in response to TME stressors may also be a potential method for tumor therapy (88).

From the above, cancer cells may engage in a “metabolic tug-of-war” with immune cells in the tumor. Overlapping MR of cancer and immune cells affects anti-tumor immune responses. On the one hand, in the TME, the scarcity of nutrients and oxygen forces immune cells to undergo MR to adapt to harsh conditions; on the other hand, cancer-induced immune cell metabolic disorders can weaken its anti-cancer properties and increase its immunosuppressive properties (89).

#### 4.2.3 The effect of MR on the treatment of CA

Drug resistance is a thorny problem that affects the cancer therapeutic effect. MR can mediate outcomes of CA treatment. In addition to inducing multidrug resistance, lipid metabolism also reverses multidrug resistance by using lipid analogues and reusing lipid-targeted drugs reprogramming the lipid composition of drug-resistant cells so that they are sensitive to drugs (90).

##### 4.2.3.1 Chemotherapy

The promotion of the Warburg effect is related to the low benefit of adjuvant chemotherapy in colorectal cancer (91). The increase in glutamine catabolism is the fundamental mechanism for inducing hypoxia in PDAC cells, leading to chemotherapy resistance (92). Crosstalk between MUC1 and HIF-1α signaling renders pancreatic cancer resistant to gemcitabine by inducing anabolic glucose metabolism (27). Yes-associated protein 1 controls the GLUT3-dependent glucose metabolism of TAMs, which enhances resistance to 5-fluorouracil in gastric cancer (93). NKX2-8 deletion-reprogrammed fatty acid metabolism contributes to chemoresistance and Perhexiline may be a potential tailored treatment for patients with NKX2-8-deleted epithelial ovarian cancer (94).

##### 4.2.3.2 Immunotherapy

Repurposing of drugs targeting cancer metabolism might synergistically enhance immunotherapy via metabolic reprogramming of the TME (95). Nanomedicines can enhance the efficacy of cancer immunotherapy by regulating lactic acid metabolism and reprogramming the immunosuppressive TME (96). Lipid metabolism reprogramming can prevent effector T cell aging and enhance tumor immunotherapy (97). The metabolic intervention of tumor-infiltrating immune cells can provide new opportunities for breaking drug resistance and improving immunotherapy (98).

##### 4.2.3.3 Radiotherapy

MR may provide new ideas for improving cancer radioresistance. Yu et al. (99) summarized the role of metabolic changes in radioresistance and proposed therapeutic targets of targeted metabolism to improve radiotherapy. Dichloroacetate (DCA) is a specific inhibitor of PDK, which can enhance the production of ROS. Although it has been described as a drug that promotes OXPHOS, DCA can also increase hypoxic radiation response (100). The role of the Warburg effect in redox homeostasis and DNA damage repair are two key factors leading to radioresistance. Furthermore, the metabolic involvement of cancer stem cells in radioresistance may be the root main cause of tumor recurrence (101).

##### 4.2.3.4 Targeted therapy

A study (102) has shown that intrinsic and escaped anti-angiogenic drugs induced tumor hypoxia initiates the MR of fatty acid and increases the uptake of free fatty acids (FFAs) that stimulate cancer cell proliferation. Targeting lipid metabolism is an emerging strategy to enhance the efficacy of anti-HER2 therapies in HER2-positive breast cancer (103). The resistance of breast cancer cells to lapatinib may be related to phosphorylation-mediated glycolysis reprogramming (104). Metabolic flexibility confers erlotinib resistance to PC by up-regulating glucose-6-phosphate dehydrogenase (105).

#### 4.2.4 The underlying mechanisms of MR

##### 4.2.4.1 Gene regulation and MR

Oncogenic KRAS may maintain pancreatic tumors by regulating anabolic glucose metabolism (18). Mutant RAS-mediated signaling pathways drive the initiation, maintenance, and progression of tumors by reorganizing cell metabolism and the TME (106). Myc regulates the transcriptional program that stimulates mitochondrial glutamine decomposition and leads to glutamine addiction (39). Tumor suppressor protein p53 has been shown to restore the Warburg effect and hurt the carcinogenic metabolic adaptation of cancer cells (107). Mutant p53 promotes breast cancer growth by maintaining serine-glycine synthesis and essential amino acid uptake (108). Activation of p53 is a promising therapeutic strategy that can reprogram tumor glucose metabolism to cell death (107). Loss of tumor suppressor PTEN expression is associated with PI3K pathway-dependent MR in hepatocellular carcinoma (109). The loss of tumor suppressor LKB1 can promote the MR of cancer cells through HIF-1α (110). Histone deacetylase SIRT6 is a tumor suppressor that controls cancer metabolism and inhibits cancer metabolism (111). Nuclear factor erythroid 2(NRF2) can activate metabolic reconnection and increase the pathway involved in glutamine metabolism, which inhibits the chemotherapy resistance of KRAS mutant pancreatic cancer (112). HIF-1 plays a key role in the MR of cancer by activating gene transcription encoding glucose transporters and glycolytic enzymes (113).

##### 4.2.4.2 Epigenetics and MR

Noncoding RNAs (ncRNA), including microRNA (miRNA), long noncoding RNA (lncRNA), and circular RNA (circRNA), can regulate the MR of cancer cells (114) and link MR to the tumor immune microenvironment (115). NcRNAs are widely involved in the rewrite of tumor metabolism. For instance, HIF-1α-stabilized lncRNA encapsulated in extracellular vesicles of TAMs can regulate aerobic glycolysis in breast cancer cells (17). LncRNA CCAT1 promotes the progression of gastric cancer through PTBP1-mediated glycolysis enhancement (116). The most common short ncRNA is microRNA, which regulates tumor metabolism by acting on metabolism-related pathways and targeting metabolism-related enzymes and proteins, thus participating in cancer progression (117). MiRNA is involved in the chemoresistance of cancer cells by regulating metabolism and can predict clinical response (56, 57). Hypoxia-induced miR-214 expression can promote tumor cell proliferation and migration by enhancing the Warburg effect in gastric cancer cells (118). CircRNA is an emerging regulator of cancer glucose metabolism. CircRPN2 can inhibit aerobic glycolysis and metastasis of hepatocellular carcinoma (119).

##### 4.2.4.3 Extracellular vesicles and MR

Extracellular vesicles (EVs) can promote tumor progression and metastasis, and drug resistance by regulating cancer cell metabolism (120). Exosomes are a subset of EVs that contain many components, not only regulating extracellular communication with cancer cells but also regulating extracellular communication with stromal cells. In the hypoxic and acidic microenvironment caused by rapid tumor growth, tumor cells tend to release more exosomes. Exosomes and their cargoes mediate MR in the TME (121). Exosomes can transfer molecules with biological functions to recipient cells. Recipient cells play an important role in tumor progression and TME by rewriting the metabolic processes of tumor cells and environmental stromal cells (122). Exosomes play a role in chemotherapy resistance by regulating the glucose and lactic acid metabolism of cancer cells and leading to MR of metabolic pathways (123). Tumor-derived exosomes can drive immunosuppressive macrophages in the pre-metastatic niche through glycolytic dominant MR (124). Cancer-derived exosome HSPC111 may promote liver metastasis of colorectal cancer by reprogramming lipid metabolism in CAFs (81).

##### 4.2.4.4 Gut Microbiota and MR

Gut fungi enhance the immunosuppressive function of myeloid suppressor cells by activating PKM2-dependent glycolysis to promote colorectal tumorigenesis (125). Microbiota-derived SSL6 increases the sensitivity of hepatocellular carcinoma to sorafenib by downregulating glycolysis (126). Microbial short-chain fatty acids modulate CD8+ T-cell responses and improve cancer immunotherapy (127). Butyrate suppresses colon cancer cell proliferation by targeting PKM2 and MR (128). *Lactobacillus iners* in the TME can alter tumor metabolism and lactate signaling pathways, leading to therapeutic resistance (129). *Akkermansia muciniphila* may play an anticancer role by interacting with tumor microbiota and reprogramming tumor metabolism in mice (130). The inflammatory tissue environment favors the disruption of MR, which is usually characterized by the massive proliferation of specific bacterial species that can utilize more abundant nutrients in the gut (131).

##### 4.2.4.5 Signal transduction pathway and MR

The common MR pathways include Hippo and JAK-STAT signaling pathways, as well as PI3K/AKT/mTOR signaling pathway, AMPK signaling pathway, and so on. The ubiquitous growth factor-regulated PI3K-AKT signaling network has a variety of downstream pathways for cell metabolism by directly regulating nutrient transporters and metabolic enzymes or by controlling transcription factors that regulate the expression of key metabolic components (35). Salt-inducible kinase 2 (SIK2) may promote MR of glucose through the PI3K/AKT/HIF-1α pathway and Drp1-mediated mitochondrial fission in ovarian cancer (132). MARK2/4 may promote the Warburg effect and cell growth of non-small cell lung cancer through AMPKα1/mTOR/HIF-1α signaling pathway (133). ROS/PI3K/Akt and Wnt/β-Catenin signal transduction activates HIF-1-induced MR and confers 5-fluorouracil resistance in colorectal cancer (134). Metabolic classification suggests the GLUT1/ALDOB/G6PD axis as a therapeutic target in chemotherapy-resistant pancreatic cancer (135).

#### 4.2.5 Modulation of MR for prevention and treatment of CA

Modification of MR in CA management is of great importance because it not only prevents the formation and progression of CA but also improves the clinical efficacy of cancer patients and reduces adverse events (136). Currently, MR intervention mainly includes the following aspects:

##### 4.2.5.1 Targeting metabolism

Targeted MR may serve as an important therapeutic method for cancer, including targeted glucose metabolism, targeted lipid acid metabolism, targeted amino acid metabolism (137), targeted lactate metabolism (138), targeted mitochondrial metabolism (139), targeted T cell metabolism (140) and targeted macrophage metabolism (85). For example, simultaneous inhibition of multiple glycolytic enzymes (PDK1 and LDH-A) is a promising new treatment for lung adenocarcinoma (141). The reversible conversion of lactate to pyruvate is catalyzed by the enzyme lactate dehydrogenase (LDH). The LDH-A isoform is considered a promising target for the treatment of various types of cancer due to its key role in the Warburg effect (142). Inhibition of LDH-A can induce oxidative stress and inhibit tumor progression (143). The 6-phosphogluconate dehydrogenase (6PGD) blockade can generate CD8+ effector T cells with enhanced antitumor function (144). FASN can be used as a therapeutic target for brain metastasis of breast cancer (145). Furthermore, targeted MR may overcome therapy resistance. For instance, targeted metabolic symbiosis may prevent resistance to antiangiogenic therapy (146). Targeted neoadjuvant chemotherapy-induced MR in PC may promote chemotherapeutic response (147).

##### 4.2.5.2 Dietary interventions

Dietary modification exerts a wide range of biological effects by exploiting the ability of dietary restrictions. For example, the ketogenic diet may target tumor metabolism and influence tumor treatment and prognosis (148). Fasting improves the therapeutic response in hepatocellular tumors through p53-dependent metabolic synergism (149). Neutralization of the acidic TME by alkalinizing agents such as bicarbonate resulted in suppression of cancer progression and potential benefit in anticancer drug response (150). Preclinical data show that increasing serum buffer capacity can neutralize the acidic TME and inhibit local invasion and proliferation, which may be synergistic with the effects of chemotherapy and immunotherapy agents (151). A glucose-restricted diet can improve host pulmonary immune responses and suppress lung tumor growth (152). Regulation of amino acid metabolism through dietary interventions may improve anticancer drug resistance (153).

##### 4.2.5.3 Pharmacologic interventions

Common drugs that interfere with MR include Melatonin and Metformin. Melatonin could antagonize HIF-1α-controlled aerobic glycolysis through ROS scavenging (154) and synergistically inhibit cell growth with sunitinib in renal carcinoma cells by reversing the Warburg effect (155). Melatonin modulates the Warburg effect and alters the morphology of hepatocellular carcinoma cell lines resulting in reduced viability and migratory potential (156). Likewise, Metformin can attenuate hepatoma cell proliferation by decreasing glycolytic flux via the HIF-1α/PFKFB3/PFK1 pathway (157) and overcome MR-induced resistance of skin carcinoma to photodynamic therapy (158). In addition, targeting metabolism with natural products may be a new strategy for cancer treatment. For instance, phytochemicals can effectively inhibit aerobic glycolysis in gastric cancer cells, suppress cell proliferation and migration, and promote apoptosis (159). Traditional Chinese Medicine can ameliorate the MR of cancer cells through its multiple pharmacological effects (160).

##### 4.2.5.4 Nanomedicine systems

Nanotechnology holds great promise for controlling cancer treatment and enhancing tumor hypoxia (161). Nanotherapeutic approaches show promising potential in altering the glycolytic metabolism of tumors (162). Glycolytic inhibitors enhanced the activity of paclitaxel and their nanoencapsulation increased their delivery in a lung cancer model (163). Nanoparticles can mediate the lipid MR of T cells in TME for immunometabolic therapy, which is expected to become a new immunometabolic therapy (164). Supramolecular nanotherapy can reprogram TAMs to inhibit tumor growth (165). In addition, the development of many nanocarriers and targeted glycolysis for the treatment of tumors has been extensively investigated to improve the therapeutic efficacy (166).

### 4.3 Limitations of the research

While bibliometrics offers valuable insights into the quantitative aspects of scholarly communication, it is crucial to approach its results critically and consider these limitations when using it as a tool for evaluating research output and impact. First, bibliometrics often overlook the qualitative aspects of scholarship, such as the relevance, quality, and originality of the research content. By solely focusing on numerical indicators, such as citation counts or publication rates, bibliometrics may not capture the full intellectual value and significance of a work. Second, the commercialization of academic publishing can influence bibliometric outcomes. Journals that operate on a profit basis might prioritize topics that attract more citations or readership, potentially distorting the academic discourse. Several factors can impact the Np by a journal over time, including special issues or calls for papers which often lead to a surge in submissions and publication, efficient manuscript handling and shorter review cycles that facilitate increased publication output, as well as open access policies that enhance accessibility and visibility of research, thereby potentially boosting submissions. Third, highly cited papers are not always the most influential or groundbreaking; they may simply be in a more popular or established research area. Finally, relying solely on a single database for bibliometric analysis may introduce biases in coverage, language, perspectives, and accessibility.

## 5 Conclusion

Over the past two decades, MR/CA research had experienced rapid growth, with significant contributions from researchers in China and the United States. *Frontiers in Oncology* published the highest number of papers, while *Cell Metabolism* received the most citations. Highly cited papers were primarily published in *Cancer Cell*, *Nature*, *Cell*, *Science*, and *Cell Metabolism*. The University of Texas System, Chinese Academy of Sciences, and Fudan University were the most productive institutions. Deberardinis Ralph J and Chiarugi Paola were the most prolific authors. Current research topics include MR in tumorigenesis and progression, MR of tumor cells and the TME, the impact of MR on cancer treatment, underlying mechanisms of MR, and modulation of MR. Future MR/CA research may focus on topics such as the tumor microenvironment, lipid MR, circular RNA, long noncoding RNA, exosomes, prognostic models, and immunotherapy. In short, this study is significant as it quantitatively analyzes academic literature to reveal patterns, trends, and influences in MR/CA research, which aids in understanding research evolution and informing future scholarly endeavors in the development trend of MR/CA, as well as guiding future research efforts aimed at improving cancer management strategies.

## Data Availability

The original contributions presented in the study are included in the article/**Supplementary Material**. Further inquiries can be directed to the corresponding authors.
