# Supplementary material for: Current hotspots and trends in cancer metabolic reprogramming: a scientometric analysis
Source: Front Immunol. 2024 Nov 11;15:1497461. doi: 10.3389/fimmu.2024.1497461 (PMC11586341; doi:10.3389/fimmu.2024.1497461)
Supplement: Supplementary file 1 [file DataSheet1.docx]

Search quires and refinement procedure:

Step 1: #1 TI OR AK = (“Tumor*” OR “Tumour*” OR “Antitumor” OR “Anticancer” OR “Cancer*” OR “Neoplasia*” OR “Neoplasm*” OR “Malignanc*” OR “Carcinoma*” OR “Oncolog*” OR “Melanoma*” OR “Lymphoma*” OR “Scrcoma*” OR "Adenocarcinoma*" OR "Leukemia*" OR "Myeloma*" OR "Blastoma*" OR "Glioma*" OR "Carcinogen*" OR "Oncogen*")

Step 2: #2 TI OR AK = (“Metaboli* Re*programming*” OR “Metaboli* Remodel*ing” OR “Metaboli* Rewiring”)

Step 3: 3: #1 AND #2 and Science Citation Index Expanded (SCI-EXPANDED) (Web of Science Index) and Meeting Abstract or Editorial Material or Early Access or Book Chapters or Correction or Letter or Proceeding Paper or Retracted Publication or News Item or Retraction (Exclude – Document Types) and 2024 (Exclude – Publication Years)

Chaining search:

https://webofscience.clarivate.cn/wos/woscc/summary/061df366-3de1-43ab-b63a-99388357b77e-e30fb8d6/times-cited-descending/1
